# From Failure to Success: Lithotripsy Transforms Lead Extraction in a Same‐Patient Case

**DOI:** 10.1111/jce.70052

**Published:** 2025-08-13

**Authors:** Ghazaleh Goldar, Edward M. Powers

**Affiliations:** ^1^ Division of Cardiovascular Diseases University of Iowa Iowa City Iowa USA

**Keywords:** infective endocarditis, lead extraction, shockwave intravascular lithotripsy

## Abstract

**Introduction:**

Cardiac implantable electronic device infections often necessitate extraction, which can be complicated by calcified adhesions, leading to increased procedural risk and reduced efficacy. Given these challenges, intravascular lithotripsy (IVL) has been proposed as a tool for lead extraction to aid in overcoming calcification. This report presents the first reported case where shockwave IVL was successfully employed to extract a severely calcified lead system after an initial extraction attempt failed due to extensive calcification.

**Clinical Background:**

A 58‐year‐old woman with a dual‐chamber pacemaker and recurrent MSSA bacteremia was found to have extensive calcification around her leads. An initial TLE attempt failed due to extensive calcifications.

**Intervention:**

Three months later, given ongoing bacteremia, IVL was used during a repeat percutaneous extraction attempt. Lithotripsy pulses were applied via balloon catheter to disrupt calcified adhesions, enabling sheath advancement and lead mobilization.

**Results:**

Both atrial and ventricular leads were successfully extracted without major complications. The use of IVL directly enabled success where prior methods had failed.

**Conclusion:**

This is the first reported case of successful CIED lead extraction using IVL following a failed initial attempt due to severe calcification. This case illustrates IVL's potential in overcoming specific high‐risk anatomic challenges.

## 1 Introduction

Cardiac implantable electronic device (CIED) infections are associated with significant morbidity and mortality and are a Class I indication for transvenous lead extractions (TLE) in patients with CIEDs [1]. However, TLE can be challenging, especially with prolonged lead dwell times, where exposure to foreign bodies has led to heavy fibrosis and calcification. Current technologies—such as telescoping sheaths, laser energy, mechanical cutting tools, and snaring techniques—are highly effective at addressing fibrosis and light calcification but can fail when attempting to liberate leads from heavy calcification. Additionally, extraction from severely fibrosed and calcified leads increases the risk of catastrophic injury, such as vascular tears and myocardial avulsion [2].

The potential for such complications can deter clinicians from performing necessary extractions, especially in cases of infection, leading to poorer patient outcomes. The risk of complications in particularly difficult extractions is a deterrent for elective extractions even when guidelines recommend extraction due to the long‐term morbidity associated with lead retention [1]. Given these challenges, intravascular lithotripsy (IVL) has been proposed as a tool for lead extraction to aid in overcoming calcification.

In an initial series of extractions utilizing IVL, a retrospective analysis by Latembi and Anderson found that patients who received IVL had shorter extraction times compared to those who did not receive this pre‐treatment [3]. However, the study did not reveal differences in the rate of clinical success between the two groups. It is important to note that this comparison was observational and did not account for potential confounding factors.

In this case, we discuss the first reported case of using vascular lithotripsy to successfully extract a severely calcified system in a patient where a prior attempt to extract without IVL had failed due to an inability to disrupt severe intravascular calcification. This case highlights the effectiveness of using IVL during the extraction of high‐risk leads.

## 2 Case Description and Intervention

**Figure 1:**
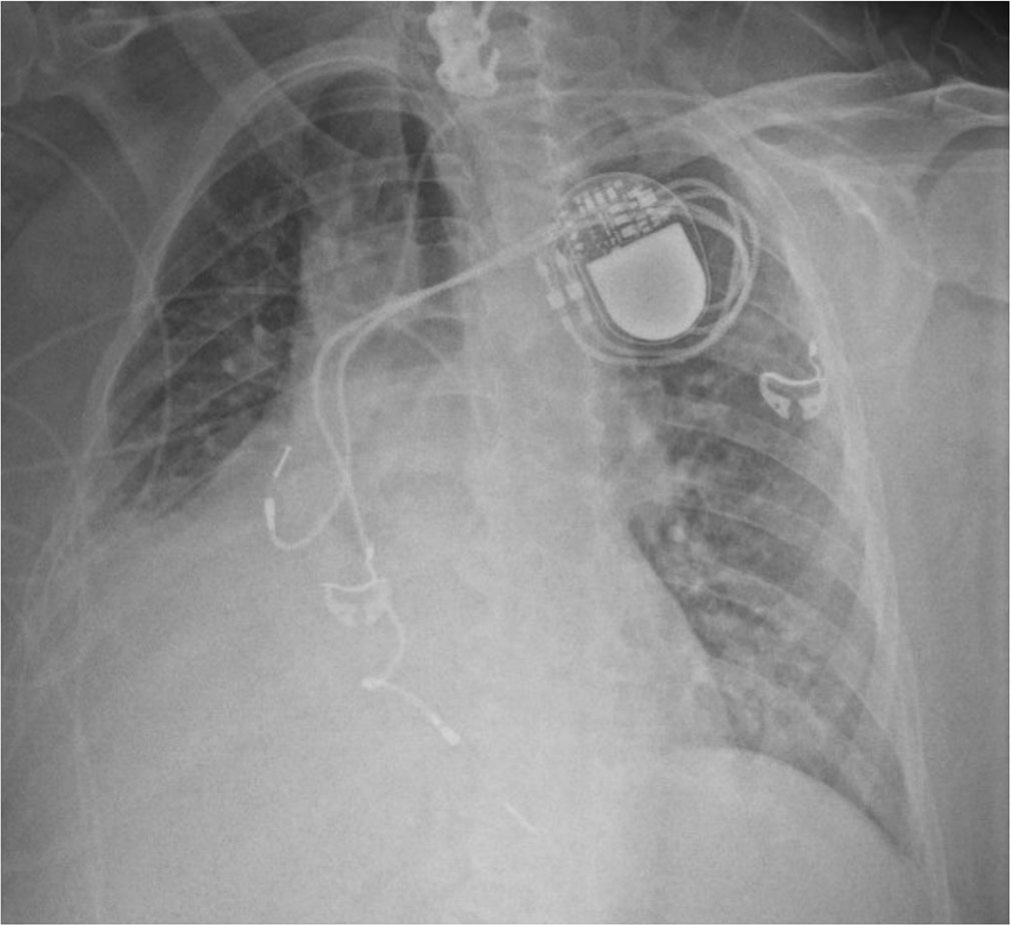
Baseline chest radiograph demonstrating the patient's dual‐chamber cardiac device in situ, before any lead extraction attempts.

**Figure 2:**
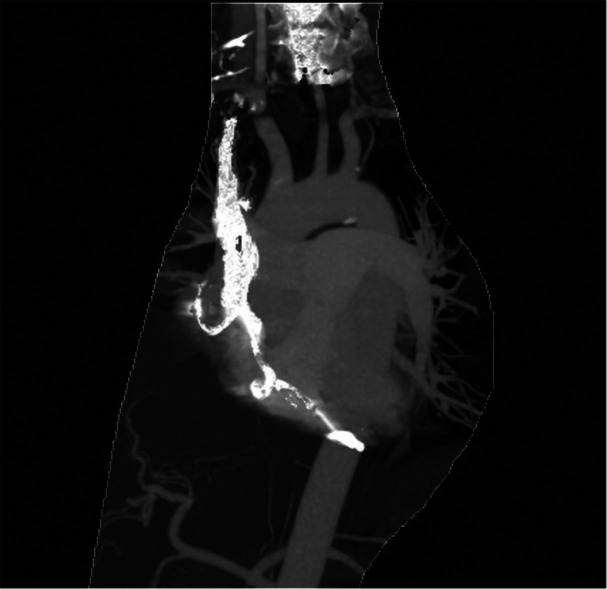
Chest CT scan demonstrating severe calcification surrounding the cardiac implantable electronic device leads. This imaging was performed to assess the extent of calcification and inform lead extraction planning, highlighting areas of significant calcified encasement.

The patient is a 58‐year‐old female with a reported history of tachy‐brady syndrome, diagnosed in 2001 at the age of 35, leading to placement of a dual‐chamber pacemaker. The device was a Medtronic, with lead models 5524 M and 5024 M for the right atrium (RA) and right ventricle (RV), respectively. Both leads were CapSure passive fixation silicone leads with an outer diameter of 8.5 French. A generator change was performed in 2017. Her medical history was also notable for neurogenic bladder, complicated by recurrent urinary tract infections, and stage II chronic kidney disease. She presented with persistently positive blood cultures growing methicillin‐sensitive *Staphylococcus aureus* (MSSA) without a clear source. Electrophysiology and cardiothoracic surgery were consulted for device extraction. A chest X‐ray and computed tomography (CT) scan was performed, the latter of which revealed severe calcification (Figures 1 and 2).

## 3 First Extraction Attempt

**Figure 3:**
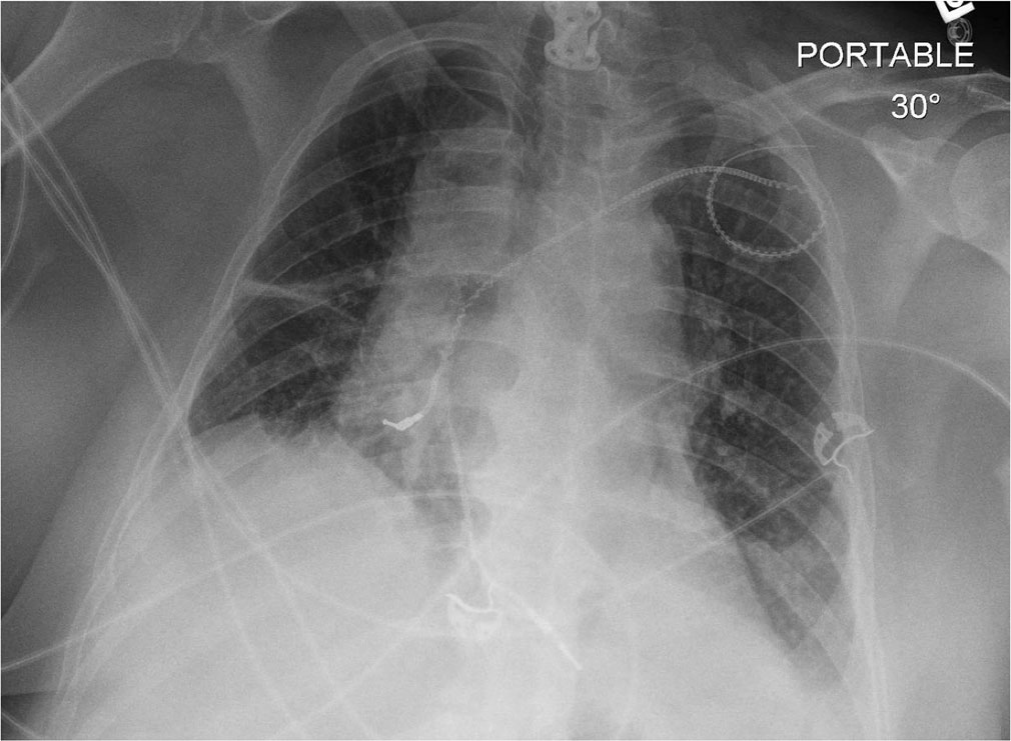
Chest X‐ray showing abandoned RV and RA leads following first attempt of extraction.

Upon opening the pocket, the lead wrap was noted to be fully embedded in a 3 mm thick block of calcification overlying the pectoral muscle. The plate of calcium and leads was liberated from the muscle, and the leads were cut away from the calcium with Mayo scissors. The leads were prepped for extraction by removing the pin and exposing the inner conductor. Locking stylets were deployed to the lead tips, and Fiberwire ties were secured on the outer insulation with a constrictor knot and two half‐hitches. The 14 Fr 80 Hz laser extraction sheath (Philips Glidelight Laser Sheath) and co‐mounted radiopaque polyurethane manual sheath (VisiSheath) were applied in an alternating fashion between the two leads due to lead‐lead binding, successfully advancing to the innominate vein. However, beyond this point, due to extensive adhesions of the lead, the laser could not be advanced further. The 11 Fr mechanical dilator sheath (Tightrail) was then advanced over the leads in an alternating fashion to the level of the superior vena cava (SVC)/RA junction before stalling. Fluoroscopic imaging demonstrated lead‐lead binding with heavy calcification at this site. Some deterioration of the RV lead was noted, so the Tightrail was exchanged for a 16 Fr laser sheath with VisiSheath and used on both leads with aggressive VisiSheath blunt dissection to disrupt the site of lead‐lead binding. No progress was made, and further deterioration of the RV lead was noted. A 13 Fr Tightrail sheath was applied next to the RA lead without advancement. The Tightrail was then applied to the RV lead, which fully transected the RV lead, leaving the proximal end embedded in the calcification at the SVC/RA junction. The sheath was then applied to the RA lead, but no progress was made. The RA lead deteriorated as well, although it remained attached via the locking stylet. The external portion of the locking stylet was cut within the proximal mechanism of the Tightrail during ongoing attempts to remove the RA lead. A bulldog lead extender was applied, and a 13 Fr Evolution RL (Cook Medical) was applied to the RA lead assembly. Again, the calcification proved too dense to advance the sheath. An attempt was made to cut the RA lead within the body at the same level as the RV lead, but this was unsuccessful. On the recommendation of the surgeon, further attempts were abandoned. The RA lead was cut to a length of 10 cm externally, secured with suture collars, and placed in a Tyrex antibiotic pouch before the pocket was closed. Figure 3 shows the chest X‐ray following the first extraction attempt. Given the patient's significant comorbidities and high risk of surgical complications, the decision was made to pursue prolonged antibiotic therapy, with the option of open extraction if infection recurs.

## 4 Second Retrieval Attempt

The patient re‐presented to a local hospital 3 months after completing antibiotics, complaining of general malaise. She was found to be bacteremic with MSSA again, and a transesophageal echocardiogram (TEE) revealed large vegetations on the leads within the RA. She was subsequently transferred to our center for further management. A discussion with cardiothoracic surgery was held regarding an open extraction; however, due to the patient's poor health and high risk of complications, they requested that we reattempt the percutaneous extraction.

The procedure was performed in the hybrid OR with a plan to proceed with open extraction if percutaneous extraction failed. After standard femoral access and preparation, a 14 Fr deflectable unidirectional long sheath and TEE were used to guide a 12 Fr Penumbra vacuum catheter to successfully remove lead vegetations that had formed on the leads and RA tissue. Next, a 0.014 wire and a 12 mm lithotripsy catheter were introduced via the 14 Fr long sheath. As shown in Supporting Information S1: Video 1, the lithotripsy balloon was inflated to 4 atm nominal pressure and rounds of 30−60 pulses were applied to the areas of interest. Additionally, the deflectable sheath was used to ensure apposition to the areas of calcification from the distal SVC to the high RA with TEE confirmation of apposition (Supporting Information S1: Video 1).

**Figure 4:**
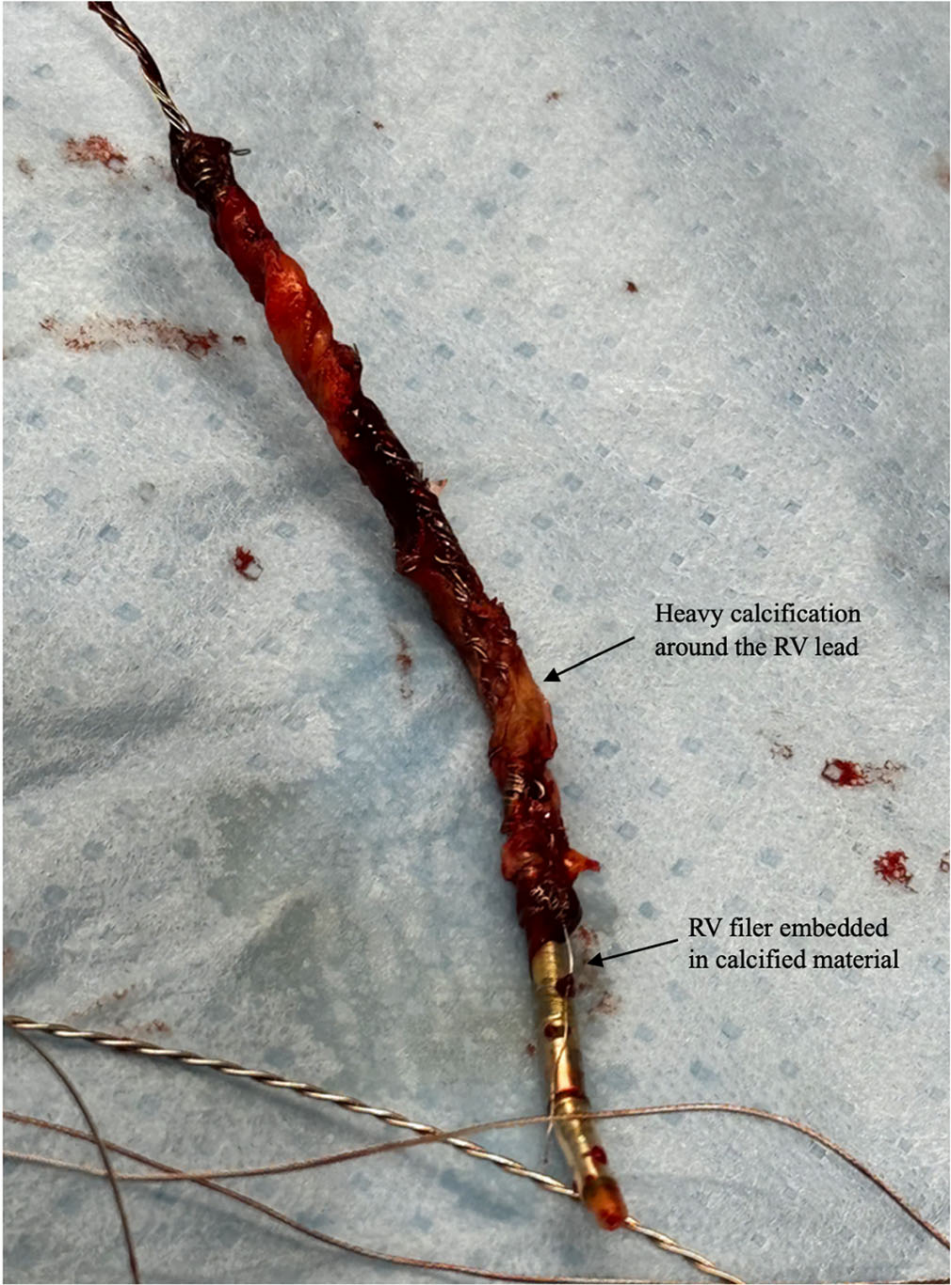
Visual inspection of the extracted RA lead reveals extensive calcification, with a fragment of a RV filer embedded in the calcified material. This finding highlights the severe calcification present at the RA/SVC junction.

Next, the severed RV lead was snared via a femoral approach using a Needle's eye snare. The lead was adhered to the atrial septum, so the system was modified to use a medium curl 8.5 Fr Agilis sheath as the inner sheath, successfully snaring the lead in the tricuspid valve/basal RV. Traction was applied with marked hypotension occurring immediately due to RV collapse, so further attempts to remove the RV lead were halted in favor of RA lead extraction. A bulldog was then attached to extend the RA lead, and a fiberwire suture was secured with two half‐hitches. The laser and VisiSheath assembly were then mounted, but they could not pass the clavicle due to fibrosis around the exposed conductors. An 11 Fr Tightrail subclavian tool was used to clear the binding to the clavicle. The 16 Fr laser and VisiSheath were reapplied to the RA lead assembly and advanced to the SVC/RA junction using a combination of laser delivery and blunt dissection, as fibrosis had formed around the exposed metal filers in the intervening 6 months. At the level of the lead‐lead binding, blunt dissection was performed, allowing easy separation of the RA and RV leads while applying gentle traction on the RV lead. The RV lead was pulled down to the low RA to clear it from the field of the RA lead, and then the VisiSheath was used to easily dissect the remaining attachments of the RA to the tissue. Attempts to remove the RA lead were unsuccessful until it was noted that an RV filer remained embedded in the calcification on the RA lead and was connecting the leads. The VisiSheath was used to sever the filer, and then the RA lead was easily removed from the body. The RV lead was then successfully removed with moderate tension from manual traction applied from below. Following extraction, visual inspection of the leads revealed calcified lesions on both the RA and RV leads that corresponded with the level of the SVC/RA junction, as well as a fragment of an RV filer originating in the block of calcium on the RA lead (Figure 4).

## 5 Conclusion

This case report illustrates the potential benefits of using shockwave IVL for the extraction of severely calcified leads. The successful application of IVL in this particularly challenging scenario—where a prior extraction attempt had failed due to dense calcification at the SVC/RA junction—highlights its utility as an effective and valuable adjunctive tool in complex lead extractions.

While the report by Latanich and Anderson demonstrated the feasibility of IVL to facilitate lead extraction, our case is, to our knowledge, the first published example of a redo extraction procedure in which a prior attempt failed due to extensive calcification and a subsequent attempt succeeded specifically with the addition of IVL. This distinction is important because it directly addresses a common criticism of IVL in this setting—namely: “How do we know that lithotripsy makes a difference?” By demonstrating procedural success in the same patient after a failed attempt without IVL, our case helps control for interpatient variability and provides compelling evidence for IVL's incremental value in overcoming severe calcific burden.

Although prior observational studies, including those by Latanich and Anderson, suggest that IVL may reduce active extraction time without necessarily improving overall success rates, this case suggests that in the most difficult scenarios, IVL may in fact enable procedural success where it would not otherwise be achievable.

It is also worth mentioning that in addition to IVL, the traction applied to the RV lead during RA lead dissection through the femoral‐based extraction techniques may have played a role in the successful separation of the components during the second attempt. However, the site of stalling of extraction tools on the RA lead was proximal to the start of the RV lead fragment, so the role of traction is uncertain. Further studies with controlled designs are needed to validate these findings, assess long‐term outcomes, and develop standardized protocols for the use of IVL in lead extraction procedures.

## Conflicts of Interest

The authors declare no conflicts of interest.

## Supporting information


**Video 1:** Panel (A) is a video demonstrating the lithotripsy technique used for SVC/RA junction preparation. The lithotripsy balloon was inflated to nominal pressure, and 30–60 pulse cycles were delivered at targeted segments. Panel (B) is a figure showing how a deflectable sheath facilitated precise apposition to the calcified regions extending from the distal superior vena cava to the high right atrium.

## Data Availability

The data supporting the findings of this study are available upon request from the authors. Please contact Ghazaleh Goldar at goldar.ghazal@gmail.com for access to the data. Access may be subject to restrictions based on confidentiality agreements or data use policies.
